# Genome-Wide Association Analysis of Over 170,000 Individuals from the UK Biobank Identifies Seven Loci Associated with Dietary Approaches to Stop Hypertension (DASH) Diet

**DOI:** 10.3390/nu14204431

**Published:** 2022-10-21

**Authors:** Olatz Mompeo, Maxim B. Freidin, Rachel Gibson, Pirro G. Hysi, Paraskevi Christofidou, Eran Segal, Ana M. Valdes, Tim D. Spector, Cristina Menni, Massimo Mangino

**Affiliations:** 1 Department of Twin Research and Genetic Epidemiology, King’s College London, London SE1 7EH, UK; 2 Department of Nutritional Sciences, King’s College London, London SE1 9NH, UK; 3 Department of Computer Science and Applied Mathematics, Weizmann Institute of Science, Rehovot 7610001, Israel; 4 Academic Rheumatology Clinical Sciences Building, Nottingham City Hospital, University of Nottingham, Nottingham NG5 1PB, UK; 5 NIHR Biomedical Research Centre at Guy’s and St Thomas’ Foundation Trust, London SE1 9RT, UK

**Keywords:** DASH, genetic, GWA

## Abstract

Diet is a modifiable risk factor for common chronic diseases and mental health disorders, and its effects are under partial genetic control. To estimate the impact of diet on individual health, most epidemiological and genetic studies have focused on individual aspects of dietary intake. However, analysing individual food groups in isolation does not capture the complexity of the whole diet pattern. Dietary indices enable a holistic estimation of diet and account for the intercorrelations between food and nutrients. In this study we performed the first ever genome-wide association study (GWA) including 173,701 individuals from the UK Biobank to identify genetic variants associated with the Dietary Approaches to Stop Hypertension (DASH) diet. DASH was calculated using the 24 h-recall questionnaire collected by UK Biobank. The GWA was performed using a linear mixed model implemented in BOLT-LMM. We identified seven independent single-nucleotide polymorphisms (SNPs) associated with DASH. Significant genetic correlations were observed between DASH and several educational traits with a significant enrichment for genes involved in the AMP-dependent protein kinase (AMPK) activation that controls the appetite by regulating the signalling in the hypothalamus. The colocalization analysis implicates genes involved in body mass index (BMI)/obesity and neuroticism (*ARPP21, RP11-62H7.2, MFHAS1, RHEBL1*). The Mendelian randomisation analysis suggested that increased DASH score, which reflect a healthy diet style, is causal of lower glucose, and insulin levels. These findings further our knowledge of the pathways underlying the relationship between diet and health outcomes. They may have significant implications for global public health and provide future dietary recommendations for the prevention of common chronic diseases.

## 1. Introduction

In 2016, the World Health Organization (WHO) estimated that more than 1.9 billion adults were overweight or obese with alarming projections indicating that by 2030, nearly 60% of the worldwide population could be either overweight or obese [1]. Obesity is a common metabolic disease and a major risk factor for other common chronic diseases, including cardiovascular disease (CAD), type 2 diabetes (T2D), metabolic syndrome, and cancer [2,3]. Moreover, obesity has been linked to common mental disorders [4]. Since both chronic diseases and mental disorder present an enormous economic burden to society, understanding the relationships between nutrition, lifestyle, and individual health has become one of the highest priorities for public health organizations [5]. Indeed, diet interventions combined with physical activity have been shown to prevent or mitigate the risk of developing common chronic diseases and mental health disorders [5,6,7]. 

To estimate the impact of diet on individual health, epidemiological and genetic studies have so far focused on different aspects of dietary intake such as macronutrient composition, curated measures of single food intake, multivariate dietary patterns described by principal component analysis (PCA) and food liking. Five genome-wide association studies (GWAs) have been conducted on macronutrient intake [8,9,10,11], two GWAs were performed on the PCA derived from the average consumption of defined diet components (i.e., meat, fish, vegetables) [12,13] and, more recently, a large scale GWA of food liking assessed food preferences over 139 specific foods [14]. Moreover, the Neale Lab (http://www.nealelab.is/uk-biobank/ accessed on 1 April 2022) conducted GWAs of thousands single food intakes (i.e., wholemeal bread vs. all food intakes) in 361,000 unrelated individuals in UK Biobank (UKB).

However, analysing individual food groups in isolation presents some limitations since the complexity of the diet pattern as a whole is not considered [15]. Indeed, consumption of certain food groups (e.g., fruit often with vegetables, or fat with sugar) is often correlated [16] and the impacts of macronutrient composition on weight change have been particularly controversial [17]. 

Over recent decades, the research has shifted towards the analysis of dietary patterns (dietary indices). Dietary indices enable a holistic estimation of diet and account for the intercorrelations between nutrients or foods and for the possible synergic effects of nutrients [15]. We have recently reported that adherence to the Dietary Approaches to Stop Hypertension (DASH) diet decreased cardiovascular risk phenotypes [18] and we found that DASH is under strong genetic influence, with heritability estimates of 37% [19]. 

Following up on these findings [18,19], here we report the results of what, to our knowledge, is the first GWA that seeks to identify putative genetic determinants of DASH. For this purpose, we used available food questionnaire data and genotypes from 173,701 subjects of European ancestry participating in the UKB collection [20]. We applied in silico functional analyses on the identified loci to gain insights into the biological processes that potentially regulate dietary intake and conducted a Mendelian randomization analysis to provide evidence of the causal relationship between DASH and both cardiovascular risk factors and mental health disorders.

## 2. Materials and Methods

### 2.1. Study Population

We carried out a GWA analysis for DASH index that was calculated based on the UK Biobank 24 h dietary recall questionnaire [21]. In the UK Biobank, dietary intake was based on the average of a 24 h dietary recall of the previous day over 5 instances between 2009 and 2012 (Table 1) [21]. At the first instance, the questionnaire was completed on a touch screen. For the remaining 4 instances, an on-line questionnaire was sent to the participants. The average period between recalls was 6 months. Participants were questioned about whether they had eaten or drunk any of the approximately 300 commonly consumed foods and beverages in the previous 24 h, along with their amount and portion sizes.

A total of 203,581 individuals replied to one or more questionnaires. DASH diet scores were computed using the individual food intakes for each food group included in the DASH index [22] (Supplementary Materials Table S1). Scores range from 8 to 40 a higher score indicating closer adherence to a DASH dietary pattern. A total of 105,930 (60%) individuals replied to more than one questionnaire (Table 1).

**Table 1:** Baseline characteristics of the UK Biobank participants included in the GWA analysis.

	Total Participants (*n* = 173,701)
Age (years) (Mean (SD))	56.420 (7.864)
Gender	
Female	94,721 (54.5%)
Male	78,980 (45.5%)
BMI (kg/m^2^) (Mean (SD))	26.462 (4.633)
Townsend Index (Mean (SD))	−1.519 (2.424)
Smoking	
Yes	98,378 (56.6%)
No	75,323 (43.4%)
Alcohol (g/day per week) (Mean (SD))	13.971 (21.439)
Instances of 24 h FFQ answered	
1	67,771 (39%)
2	39,797 (22.9%)
3	35,567 (20.5%)
4	25,713 (14.8%)
5	4853 (2.8%)
Energy intake (kcal) (Mean (SD))	2106.596 (595.421)
Fruit (Mean Servings (SD))	3.16 (2.52)
Vegetables (Mean Servings (SD))	3.49 (3.06)
Nuts and legumes (Mean Servings (SD))	0.9 (1.06)
Whole grains (Mean Servings (SD))	4.05 (3.12)
Low-fat dairy (Mean Servings (SD))	0.66 (0.84)
Sodium (mg) (Mean (SD))	2998.9 (3046.66)
Red and processed meat (Mean Servings (SD))	1.66 (1.75)
Sweetened beverages (Mean Servings (SD))	0.47 (0.89)
DASH (Mean (SD))	24 (4.24)

SD, standard deviation; BMI, body mass index; FFQ, food frequency questionnaire; DASH, Dietary Approaches to Stop Hypertension; GWA, genome-wide association study.

For these subjects we computed the averages of their scores. We excluded from the analysis subjects who fell in each of the following category: (i) incomplete diet questionnaire, (ii) nutritional data not credible (UKB field 100,026) and (iii) low energy intake reported (energy intake < 1.1 × resting metabolic rate (RMR), with the RMR calculated by Mifflin St Jeor equation [23]). After the exclusions, we included in the GWA analysis a total of 173,701 individuals with both questionnaire and genotype data available (Table 1).

### 2.2. Genome Wide Association Analysis

UK Biobank is a large prospective cohort including genome wide genotyping, deep phenotyping and molecular data on over 500,000 individuals recruited throughout the UK between 2006–2010 [20]. Full details on genotyping, imputation and initial quality control of the genetic dataset have been described previously [24]. Briefly, UK Biobank participants were genotyped using two similar and mutually compatible SNP arrays platforms: the Affymetrix UK Biobank Axiom array and the UK BiLEVE array [24] and the imputation was performed combining Haplotype Reference Consortium (HRC) [25] and UK10K [26] reference panels. We also excluded from further analyses individuals based on (1) high SNP missingness (>2%); (2) extreme heterozygosity (±3 SD from mean heterozygosity rate); (3) withdrew their consent at the time of analysis; and/or (4) were not of European ancestry (flagged from the UKBB principal component analysis). Furthermore, we also excluded SNPs because they had: (1) minor allele frequency (MAF) ≤ 5% and/or (2) info score (imputation quality) ≤ 0.8. 

The genome wide association analysis was performed in BOLT-LMM [27] using a linear mixed model, in order to provide additional corrections for population structure and cryptic relatedness. DASH diet score was used as the outcome variable in the regression model, under the assumption of an additive model for allelic effects. In order to account for confounding effects, the regression model was adjusted for age, sex, BMI, energy intake, smoking status, age, alcohol intake as a categorical (0–5 g/d, 5–15 g/d, or >15 g/d) and the first five principal components. We also included in the association model the Townsend deprivation Index [28] (a composite measure of socioeconomic deprivation and household income) to account for the socio-economic status of the participants. Sex chromosomes were not included in the analysis.

### 2.3. Mapping and Conditional Analysis

We used the Functional Mapping and Annotation of Genome-Wide Association Studies (FUMA) web-based application (https://fuma.ctglab.nl/) [29] to identify single-nucleotide polymorphisms (SNPs) associated with DASH at genome-wide significant *p*-value (*p* < 5 × 10^−8^) that are in approximate linkage disequilibrium with each other at r^2^ < 0.1. The independence of these signals was directly assessed using the imputed genotype dosage for the lead SNPs as a covariate along with the other covariates from the primary GWA using a linear mixed model in BOLT-LMM (conditional analysis). We defined a novel locus if all the variants and the genes mapping in the identified loci were not previously reported associated with DASH by querying NHGRI-EBI GWAS Catalog [30], PhenoScanner (v2.0) [31] and Open Target Genetics [32].

### 2.4. Pathway and Colocalization Analysis

We used Multi-marker Analysis of GenoMic Annotation (MAGMA) (Version 1.8), implemented in FUMA web-based application, applying standard settings to identify the most likely causal genes [33]. The statistical threshold for the most credible gene was defined at *p* < 2.86 × 10^−6^ (0.05/17,460 analysed genes). To validate MAGMA results, we also conducted gene analysis and gene set analysis using VEGAS2 (version 0.2) [34] using the default options. In this case, the statistical threshold set at *p* < 2.55 × 10^−6^ (0.05/19,640 analysed genes). For each GWA locus, we performed a colocalization analysis with the ‘‘coloc’’ R package (Version 3.2-1, R Core Team, Vienna, Austria) [35].

We performed the “coloc” analysis using the publicly available genome-wide expression quantitative trait locus (eQTL) data from 31,684 whole-blood samples deposited on the eQTLgen portal [36,37]. We included in the analysis all cis-eQTLs (false discovery rate (FDR) < 0.05) present in both DASH GWA and eQTLgen results and mapping 1 Mb across the lead SNP of each locus. Analyses were performed using the recommended defaults prior probabilities (PP) (PP for association in the GWA (P1): 1 × 10^−4^; PP for association in the eQTL (P2): 1 × 10^−4^; and PP for association in both dataset (P12): 1 × 10^−5^).

To investigate the SNP functional relevance on DASH, we applied the Summary-data-based Mendelian Randomization (SMR) (Version 1.03) approach [38], integrating the summary results from eQTLGen Consortium [36,37] and DASH GWA. SMR applies the principles of Mendelian randomization (MR) to test the association between gene eQTLs and a trait using the most associated SNP as a genetic instrument [38]. A significant SMR test indicates that a functional variant determines both gene expression and the trait of interest via causality or pleiotropy. 

The heterogeneity in dependent instruments (HEIDI) test evaluates the existence of linkage disequilibrium (LD) in the observed association. Rejection of the null hypothesis (*P*_HEIDI_ < 0.05) indicates that the association might be due to two distinct variants in high LD. We performed SMR using the recommended default options and utilised the genotypes from 3601 independent TwinsUK samples to estimate the LD structures. To account for multiple testing, SMR *p*-values were adjusted using the Benjamini and Hochberg method [39]. Association tests with *p*SMR_FDR_ < 0.01 were considered statistically significant, while *p*SMR_FDR_ < 0.05 and ≥0.01 were considered “suggestive”.

### 2.5. Shared Genetic Architecture with Disease

Genetic correlations (r_g_) between DASH and complex traits were estimated using linkage disequilibrium score regression (LDSC) through LD-hub (Version 1.9.3) software performed using the LD-Hub online portal (http://ldsc.broadinstitute.org/) [40] that automates the computation of r_g_ between one phenotypic trait of interest and 855 diseases or other phenotypic traits whose summary-level GWAs results are deposited in the database. To account for multiple testing, the significance for the LDSC analysis was set at *p*LDSC_FDR_ < 0.01.

### 2.6. Mendelian Randomization

We performed a two-sample bidirectional Mendelian randomization analyses utilising the Generalised Summary-data-based Mendelian Randomization (GSMR) (Version 1.0.9) [41] implemented in the Genome-wide Complex Trait Analysis (GCTA) (Version 1.93.2) suite [42]. The GSMR estimate the effect and its standard error from multiple SNPs associated with the analysed traits at a genome-wide significance level. To perform the GSMR analysis we utilised the following summary statistics from genetic studies not overlapping UK Biobank: Body mass index (BMI) [43], high-density lipoprotein (HDL), low-density lipoprotein (LDL), triglycerides (TAG), total cholesterol (TC) [44], Glucose, Insulin [45], Homeostatic Model Assessment-Insulin Resistance (HOMA-IR) [46], coronary artery disease (CAD) [47] and Body Fat percentage [48]. In light of the results observed in the LDSC and the gene-enrichment analyses, we also included neuroticism [49] and educational attainment (years spent in formal education) [50] in the GSMR analysis. We included in the GSMR analysis data from the most recent meta-analyses (not including UK Biobank individuals). The summary statistics were downloaded either from the original consortia or from NHGRI-EBI GWAS Catalog [30] webpages and harmonized utilising the “snp_match” command implemented in the bigsnpr (Version 1.6.1) [51] package in R (R Core Team, Vienna, Austria). We only used SNPs on autosomal chromosomes and available in the HRC reference panel, which allowed us to estimate the linkage disequilibrium among the instrument SNPs and prune them.

The HEIDI test implemented in GSMR was used to detect and remove variants showing independent effects on both exposure and outcome (i.e., horizontal pleiotropy), because they do not satisfy the assumptions for valid instruments. The HEIDI test is more conservative than excluding SNPs that have an outlying association likely driven by locus-specific pleiotropy. GSMR is more powerful than other MR methods (i.e inverse-weighted MR (IVW-MR) and MR-Egger) because it takes account of the sampling variation of both the exposure and outcome effects [41]. GSMR also accounts for LD between the clumped SNPs. We used the genome-wide significant p-value threshold (*p* < 1 × 10^−8^) to select a minimum number of instrument SNPs (*n* > 5) to perform the GSMR analysis. Genotypes of unrelated TwinsUK cohort participants were used as reference to estimate the LD structures. To further validate the GSMR results we also conducted IVW-MR and MR-Egger analyses [52] using the TwoSampleMR (Version 0.5.5) [53] package in R (R Core Team, Vienna, Austria).

## 3. Results

**Figure 1:**
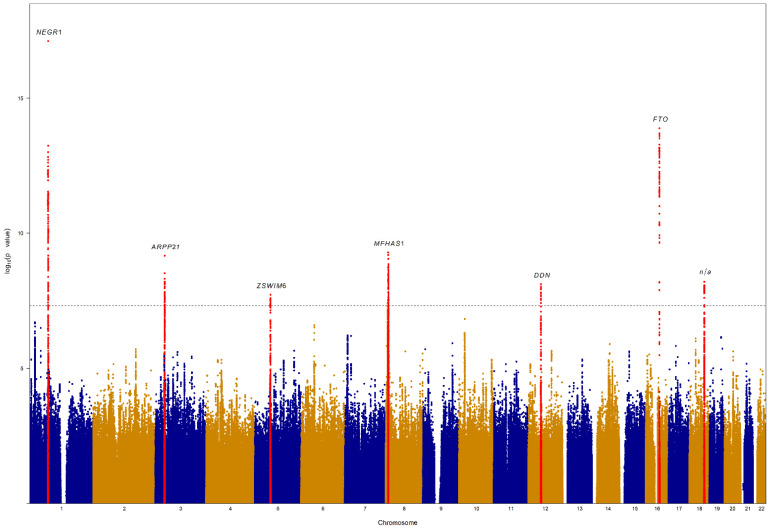
Manhattan plot of the DASH genome wide association study in UK Biobank. The x axis represents the position on each chromosome (represented with alternate colours), while the y axis represents the −log10(P) of SNPs. The dotted line indicates the genome-wide significance threshold (*p* < 5 × 10^−8^). Only SNPs with *p* < 0.1 are represented in the figure. Independent genome-wide significant variants (in red) are annotated with the genes associated with DASH based on both MAGMA and VEGAS2 analyses. No genes were associated with DASH on chromosome 18q21.32. DASH, Dietary Approaches to Stop Hypertension; SNPs, single-nucleotide polymorphisms; MAGMA, Multi-marker Analysis of GenoMic Annotation; n/a, no genes present.

We accessed the 24 h dietary recall records for 203,581 individuals from UKBiobank (Supplementary Materials Figure S1) and, after the quality controls exclusions, we calculated a DASH score for 173,701 subjects with genotype available. Full characteristics of the study population are reported in Table 1. The participants included in the final analysis had an average age of 56.4 ± 7.9, were overweight (BMI mean = 26.5 kg/m^2^ ± 4.6 kg/m^2^), had average daily energy intake of 2106.6 kcal ± 595.4 kcal and a mean DASH score of 24 ± 4.2. Half of the samples were females (54.5%) and smokers (56.6%) (Table 1). Some of the values for the food consumption reported in Table 1 reflect the typical skewed distribution observed when collecting food group data. However, the skewed distribution did not affect the DASH score because, using a point score based on the quintiles of the distribution of each food component, it has been specifically designed to address this issue [22].We tested over 12 million autosomal SNPs for association with DASH. A genomic inflation factor [54] of λ_DASH_ = 1.2 and the linkage disequilibrium score regression (LDSC) intercept of 0.997 ± 0.0099 is consistent with the expectations of polygenicity and large sample sizes [55] and indicate adequate population structure control. We observed 641 genome-wide significant associations (*p* < 5 × 10^−8^) (Figure 1), clustered within 7 distinct genomic regions (Table 2 and Supplementary Materials Figure S2).

To investigate the presence of multiple independent sources of associations within the respective associated regions, we performed conditional analyses on each of the seven regions, adjusting for the effect of the lead SNPs that were included in models as covariates. We did not detect any additional independent SNP with either significant (*p* < 5 × 10^−8^) or suggestive (*p* < 1 × 10^−7^) *p*-value.

Most associated loci point towards a shared genetic background between DASH and behaviour, food related or metabolic traits. In particular, the strongest association was detected within a 445 Kbp region on chromosome 1p31.1 (rs66495454, *p* = 7.6 × 10^−18^) (Supplementary Materials Figure S2A). The 1p31.1 locus harbours the neuronal growth regulator 1 (*NEGR1*) gene, which has previously been associated with psychiatric [56], behavioural [57], nutritional [13] and metabolic disorders [43]. We also identified one locus on chromosome 16q12.2 (rs56094641, *p* = 1.3 × 10^−14^) harbouring *FTO* (Supplementary Materials Figure S1F), best known genes influencing both nutrition and obesity [44]. Another variant (rs56331918, *p* = 6.9 × 10^−10^) mapping on chromosome 3p22.3 in the intronic region of CAMP Regulated Phosphoprotein 21 (*ARPP21*)) gene (Supplementary Materials Figure S2B) which has been associated with neuroticism [58] and BMI [59]. Our results showed a significant association between DASH and a ~3 Mb region (Chr8: 8,088,230–11,463,015; rs73195303, *p* = 5.3 × 10^−10^) on chromosome 8p23.1 (Supplementary Materials Figure S1D). Chromosome 8p23.1 locus comprises numerous genes performing functions important to the nervous system and associated with cancer and developmental neuropsychiatric disorders [60]. Finally, three additional loci were identified on chromosome 5q12.1 (rs544711163, *p* = 1.9 × 10^−8^), 12q13.12 (rs1054442, *p* = 7.7 × 10^−9^) and 18q21.32 (rs35614134, *p* = 6.3 × 10^−9^), (Supplementary Materials Figures S2C, S2E, S2F and S2G, respectively). 

Using MAGMA [33], we performed a gene-based analysis including the complete GWA results. This analysis identified nineteen genes associated (*p* < 2.66 × 10^−6^) with DASH. While most of these genes (15 out of 19) mapped within the identified loci, MAGMA analysis also found four genes which are physically distant (>250 kb) from the lead SNPs. Supplementary Materials Table S2 lists the fifteen most likely causal genes (reported by MAGMA analysis) in or near the lead SNP at each locus. Similar results were obtained when the analysis was performed using VEGAS2 (Supplementary Materials Table S3).

In order to annotate these genes in a biological context, we used the function GENE2FUNC included in FUMA online platform [29]. We observed that our GWA results were enriched for genes participating in the Activation of AMPK downstream of N-methyl D-aspartate receptors (NMDARs) pathway, even after Bonferroni multiple testing correction (*p*_adjusted_ = 2.8 × 10^−2^) (Supplementary Materials Table S4). We also find significant enrichment for genes participating in another 40 gene-sets selected from the GWA catalogue (*p*_adjusted_ ranging from 2.7 × 10^−17^ to 4.4 × 10^−2^), in particular, the “general factor of neuroticism” which was one of the most significantly enriched entries (*p*_adjusted_ = 2.7 × 10^−17^) (Supplementary Materials Table S4 and Figure S3).

Next, we proceeded with a functional characterisation of genomic variants within or near genes harboured by the identified loci. We performed a Bayesian test [35] to examine whether GWA loci co-localize with the gene eQTLs in blood. To this end, we utilised publicly available expression data from the large eQTLgen consortium meta-analysis [37]. We identified 111 genes mapping in the 8 identified loci. Seventy-two genes were excluded from the analysis because they were not available in the eQTLgen dataset (Supplementary Materials Table S5). Our COLOC-estimated posterior probabilities (PP) [35] suggested that two eQTL effects (*ARPP21* and *RHEBL1*) were probably sharing the same common causal variant (PP_H4_ ranging from 1 to 0.80) with the associated loci (Supplementary Materials Table S5). Twenty-six eQTLs overlapped with their corresponding locus, without necessarily sharing the same causal variant (PP_H3_ ranging from 1 to 0.80). The COLOC-PP for *NEGR1* (PP_H4_ = 0.68), *PRKAG1* (PP_H4_ = 0.65) and *FTO* (PP_H4_ = 0.74) eQTLs showed a suggestive probability for a causal variant shared with chromosome 1p31.1, 8p23.1 and 16q12.2 loci, respectively. Finally, the analysis of eight eQTL transcripts showed no colocalization (PP_H0_ or PP_H1_ ranging from 1 to 0.80) or failed to support any tested hypotheses (All PPs < 0.80) (Supplementary Materials Table S5).

To further evaluate whether any of the cis-eQTLs mediated the association between genetic variants and DASH, we applied SMR [38] on the GWA results. Nine genes did not have any significant eQTL SNP (*p* < 5 × 10^−8^) to be utilised as a genetic instrument and therefore were excluded from the analysis (Supplementary Materials Table S6). We observed statistically significant associations (*p*SMR_FDR_ <0.01) for fifteen genes (*p*SMR_FDR_ ranging from 1.03 × 10^−20^ to 2.06 × 10^−3^) with eight genes showing a suggestive SMR (0.05 < *p*SMR_FDR_ ≤ 1 × 10^−2^) (Table S6). The SMR test was not significant for seven genes (Supplementary Materials Table S6). We obtained similar results when using a SMR multi-SNP approach (*p*SMR_FDR_ ranging from 3.15 × 10^−19^ to 3.34 × 10^−3^) (Supplementary Materials Table S6). Next, we performed the HEIDI test to distinguish pleiotropy/causality from linkage. The HEIDI results suggested that four genes (*ARPP21, RP11-62H7.2, MFHAS1, RHEBL1*) were consistent with either pleiotropy or causality (Supplementary Materials Table S6) while for eleven genes it was not possible to distinguish between pleiotropy/causality and linkage disequilibrium (HEIDI *p* ranging from 6.8 × 10^−13^ to 4.4 × 10^−2^).

**Figure 2:**
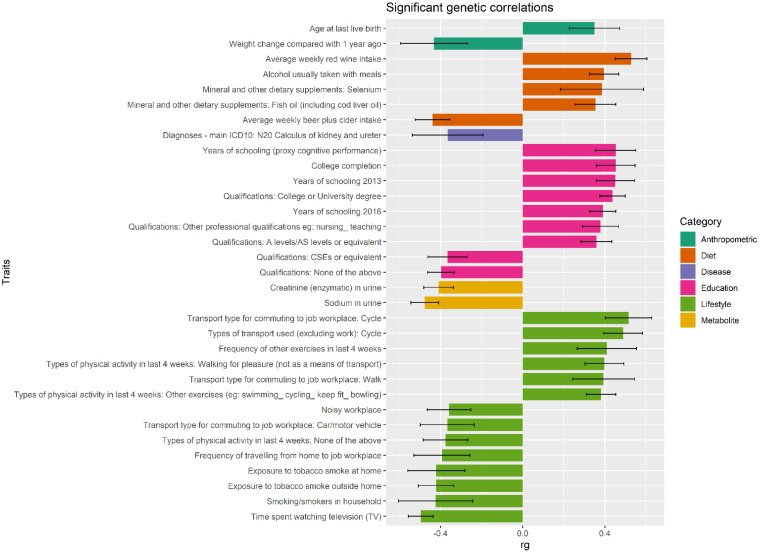
Genetic correlations (r_g_). Pairwise genome-wide genetic correlations between DASH and 855 other phenotypic traits or diseases were estimated using LD score regression (LDSC). In the figure are represented only the strongest genetic correlations (r_g_ < −0.35 and r_g_ > 0.35) (see ESI Supplementary Materials Table S7 for the report of the full results). Error bars show 95% confidence intervals, while colours represent the different categories. LD, linkage disequilibrium; ICD10, International Statistical Classification of Diseases and Related Health Problems 10th Revision; A, Advanced; AS, Advanced Subsidiar; CSE, Certificate of Secondary Education.

To compute the amount of shared genetic correlation (r_g_) between DASH and other complex traits, we performed LD score regression (LDSC) analyses on 855 other phenotypic traits or diseases [40] and observed significant genetic correlations (*p*LDSC_FDR_ < 0.01) with 193 of them (Supplementary Materials Table S7). Among traits with strong positive genetic correlations with DASH the most significant was educational attainment (“Qualifications: College or University degree”, (r_g_ = 0.44, *p*LDSC_FDR_ = 4.7 × 10^−41^). We also found significant positive genetic correlations with other measures of educational attainment (Supplementary Materials Table S7 and Figure 2). “Average weekly red wine intake” was the trait with the strongest genetic correlation (r_g_ = 0.53, *p*LDSC_FDR_ = 2.6 × 10^−39^). Among the strongest negative correlations (r_g_ < −0.35), “Time spent watching television (TV)” was the trait showing the strongest and most significant genetic correlation with DASH (r_g_ = −0.50, *p*LDSC_FDR_ = 2.3 × 10^−56^) (Supplementary Materials Table S7 and Figure 2)

We previously described the association between DASH and cardiometabolic traits [18] and next sought to explore the evidence of causality versus pleiotropy between DASH and these traits using bidirectional a GSMR [41]. In light of the newly observed LDSC and gene-enrichment evidence implicating neuroticism and educational attainment, both phenotypes were also included in the GSMR analyses.

Bidirectional GSMR strongly suggested that high DASH scores causally lower insulin (Beta _DASH->Insulin_ (standard error (SE)) = −0.041 (0.01); *p*_GSMR_ = 3.86 × 10^−4^) and glucose levels (Beta _DASH->Glucose_ (SE) = −0.036 (0.01); *p*_GSMR_ = 8.82 × 10^−3^) (Supplementary Materials Table S8 and Figure S4A,B). Our results also provided evidence of a causality of high DASH score on increased educational attainment (Beta _DASH->Educational Attainment_ (standard error (SE)) = 0.101 (0.02); *p*_GSMR_ = 2.15 × 10^−8^) (Supplementary Materials Table S8 and Figure S4C). We did not find evidence for a causal role for insulin (*p*_GSMR_ = 7.82 × 10^−2^) and glucose (*p*_GSMR_ = 0.821) levels on DASH score (Supplementary Materials Table S8). We could not test reverse causality of educational attainment on DASH because there were not enough independent instrument variants (*n* < 5) to perform the GSMR analyses. For CAD, neuroticism score, body fat percentage, HOMA-IR, triglycerides, LDL, HDL and total cholesterol levels the GSMR analyses were not significant when considering DASH as exposure (*p*_GSMR_ = 0.123, *p*_GSMR_ = 0.451, *p*_GSMR_ = 0.634, *p*_GSMR_ = 0.127, *p*_GSMR_ = 0.854, *p*_GSMR_ = 0.397, *p*_GSMR_ = 0.517 and *p*_GSMR_ = 0.059, respectively) (Supplementary Materials Table S8). We also observed a causal relationship between lower HDL levels (Beta _HDL->DASH_ (SE) = −0.066 (0.03) *p*_GSMR_ = 2.32 × 10^−2^), high LDL levels (Beta _LDL->DASH_ (SE) = 0.112 (0.03) *p*_GSMR_ = 1.75 × 10^−5^), increased levels of total cholesterol (Beta _TC->DASH_ (SE) = 0.071 (0.03) *p*_GSMR_ = 8.65 × 10^−3^), increased CAD risk (Beta _CAD->DASH_ (SE) = 0.081 (0.03); *p*_GSMR_ = 2.57 × 10^−3^) and increased body fat percentage (Beta _BODY FAT%->DASH_ (SE) = 0.426 (0.15); *p*_GSMR_ = 4.83 × 10^−3^) when considering DASH as outcome (Supplementary Materials Table S8 and Figure S4D–G). We could not test reverse causality of neuroticism and HOMA-IR on DASH because there were not enough independent instrument variants (*n* < 5) to perform the GSMR analyses. Finally, for BMI, we found significant bidirectional effect with DASH (Supplementary Materials Table S8). This result may reflect the presence of shared biological pathways (vertical pleiotropy). The GSMR results are reported in full in Supplementary Materials Table S8. We obtained qualitatively similar results with other MR methods implemented in the two-sample MR R library (Supplementary Materials Table S8). As different MR methods rely on different assumptions and models of horizontal pleiotropy, the consistency of the results across different methods builds confidence in the obtained estimates.

## 4. Discussion

In this first GWA, we investigated the genetic influences on DASH on over 170,000 subjects of European ancestry and identified seven associated loci, which provide new insights into the genetic basis of this dietary pattern. By leveraging these genetic findings, we performed Mendelian randomization analyses to assess the causal relationship between DASH and health outcomes. Our results indicate that a healthy diet style may causally lead to reduced levels of glucose and insulin. The gene-based analysis revealed nineteen genes associated with DASH. We identified four genes (*ARPP21*, *RP11-62H7.2*, *MFHAS1* and *RHEBL1*) whose expressions were potentially associated with DASH due to causality or pleiotropy. Interestingly, most of these genes have been consistently associated with cardiometabolic diseases [59,61], educational attainment [50], cognitive abilities [62], neuroticism [58] and major depressive disorder [60]. These findings support the hypothesis that genes influencing dietary choice may also influence the liability to psychiatric and cardiometabolic disorder [13].

A recent UK Biobank study defined two independent diet component (DC) intakes based on the principal component (PC) of UK Biobank generic diet questionnaire and identified a number of genetic loci associated with either DC1 (a meat-related diet) or DC2 (a fish/plant-related diet) [13]. Moreover, utilising the same UK Biobank generic diet questionnaire, May-Wilson et al. identified the genetic determinants of food liking [14]. Two markers reported in our study overlap with the variants associated with either DC1 (rs66495454 on chromosome 1p31.1) or DC2 (rs56094641 on chromosome 16q12.2). Specifically, the rs66495454 A allele that is associated with a lower DASH score, indicating a propensity to lower diet quality also increased processed meat intake [13] as well as red meat and beef steak liking [14] (all negative components of the DASH diet).

Similarly, the variant (rs56094641) on chromosome 16q12.2 is associated with lower DASH score as well as lower non-oily fish intake/liking [13,14]. Similar results were also reported by Cole et al.’s analyses [12] of measures of single food intake (FI) in UK Biobank (1p31.1 and 16q12.2, harbouring *NEGR1* and *FTO* genes, respectively). These two genes have been consistently associated with BMI [43,63] obesity and cardiometabolic diseases [43]. Although we were not able to distinguish between causal effect and linkage disequilibrium, the SMR analysis on chromosome 1p31.1 locus showed that decreased *NEGR1* expression levels are associated with lower DASH score. Decreased expression level of *Negr1* in murine periventricular hypothalamic areas lead to an increase in body weight [64]. Altogether, these results are consistent with previous observation that increased red/processed meat consumption is mostly responsible for the association between DASH and increased cardiometabolic disease risk [18] and may indicate a very complex genetic relationship between DASH and obesity.

Gene-set enrichment analyses provide evidence that the genes annotated to the variants associated with DASH participate in the “Activation of AMPK downstream of NMDARs” pathway. The AMP-dependent protein kinase (AMPK) is highly expressed in the hippocampus [65] and is activated when AMP and ADP levels in the cells rise due to a variety of physiological stresses, such as increased ghrelin levels, glucose deprivation and exercise [66]. AMPK is one of the signalling components of the Neuropeptide Y (NPY) network, which is the master regulator of the appetite signal in the arcuate nucleus-paraventricular nucleus (ARC-PVN) of the hypothalamus [67]. Intracerebroventricular administration of a pharmacological AMPK activator (AICAR) in murine experiments stimulates food intake and weight gain [68]. 

Our study should be interpreted in the context of the following limitations. First, our SMR analyses is based on cis-eQTL effects estimated in peripheral blood because the currently available brain eQTL studies have very limited statistical power due to their small sample sizes. However, Ting Qi et al. demonstrated that, when the genes are expressed in both brain and blood, then using cis-eQTL effects estimated in blood as proxies of those in brain increase the power to identify putative functional genes for brain-related complex traits and diseases [69]. Additionally, similar to other UK Biobank dietary studies [12,13,14], we calculated the DASH score using the self-reported questionnaire data. Single 24-hr recalls are unlikely to capture episodic food of some items included in the DASH score (nuts, legumes). However, more than 60% of the participants included in this study answered to two or more questionnaire. Therefore, by averaging multiple recalls from participants, it is more likely that they represent the individual habitual intake. Finally, large biobanks are well powered to discover common variant associations. However, replicating their findings is one of the main issue that has been recently discussed [70]. Indeed, while using data derived from the same biobank, studies analysing similar traits (same phenotype but different modelling and phenotype definition) have solved this problem adopting different solutions [71,72]. As suggested by Huffman [70] we performed a range of secondary analyses (functional annotation, pathway analysis, eQTL and colocalization) utilizing publicly available datasets. We presented a number of orthogonal biological evidences which may be considered in the same vein as statistical validation [70]. Using different phenotype definition and analysis model, some of the findings described in this study overlap with previous observations [12,13,14]. The consistency of the results across different studies builds confidence in our findings and may represent a form of validation [70]. However, although our secondary analyses pointed towards plausible genes/pathways and some of the loci identified in this study overlap with previous observations in UK Biobank, our findings need to be further tested in functional and interventional studies in animal models and humans to fully determine the biological mechanisms underlying DASH.

## 5. Conclusions

In conclusion, this study provides novel insights into the genetic architecture of DASH and highlighted its putative causal relationship with health outcomes. These findings extend our knowledge of the genetic pathways underlying DASH and may have significant implications for global public health providing future dietary recommendations for the prevention of common chronic diseases and mental health disorders.

## Figures and Tables

**Table 2:** GWA summary results. Seven independent genomic regions associated with DASH at genome-wide significance (*p* Value < 5 × 10^−8^).

Locus	Chromosome	Locus Starts	Locus End	Top SNP	Top SNP Position	Effect Allele	Non-Effect Allele	Effect Allele Frequency	Beta	Standard Error	*p* Value
1	1	72,511,514	72,956,535	rs66495454	72,748,567	GTCCT	G	0.38	0.126	0.01	7.60 × 10^−18^
2	3	35,778,773	35,913,342	rs56331918	35,801,168	G	C	0.28	−0.1	0.02	6.90 × 10^−10^
3	5	60,613,826	60,844,213	rs544711163	60,775,743	CT	C	0.38	−0.082	0.01	1.90 × 10^−8^
4	8	8,088,230	11,463,015	rs73195303	10,200,253	T	C	0.23	−0.105	0.02	5.30 × 10^−10^
5	12	49,385,679	49,479,968	rs1054442	49,389,320	C	A	0.37	0.085	0.01	7.70 × 10^−9^
6	16	53,797,908	53,845,487	rs56094641	53,806,453	G	A	0.40	0.111	0.01	1.30 × 10^−14^
7	18	57,732,418	57,912,226	rs35614134	57,832,856	AC	A	0.24	0.097	0.02	6.30 × 10^−9^

SNPs, single-nucleotide polymorphisms.

## Data Availability

The UKBB phenotypic data and GWA data analyzed during thecurrent study are available from UK Biobank.

## Supplementary Materials

The following supporting information can be downloaded at: https://www.mdpi.com/article/10.3390/nu14204431/s1, Figure S1: Study design and analysis workflow; Figure S2: Regional associations plots; Figure S3: Gene enrichment analysis results; Figure S4: Mendelian randomization analyses (GSMR); Table S1: Food groups included in the computation of DASH score; Table S2: Genes significant in MAGMA gene-wise analysis; Table S3: Genes significant in Vegas2 gene-wise analysis; Table S4: Gene-set Enrichment analysis (GENE2FUNC); Table S5: Co-localization analysis; Table S6: SMR results; Table S7: Linkage Disequilibrium Score Correlation analysis; Table S8: Mendelian randomization results.
